# Prevalence and Risk Factors of Heart Failure with Preserved Ejection Fraction: A Population-Based Study in Northeast China

**DOI:** 10.3390/ijerph13080770

**Published:** 2016-07-29

**Authors:** Liang Guo, Xiaofan Guo, Ye Chang, Jun Yang, Limin Zhang, Tan Li, Yingxian Sun

**Affiliations:** 1 Department of Cardiology, the First Hospital of China Medical University, Shenyang 110001, China; doctorgl@126.com (L.G.); guoxiaofan1986@foxmail.com (X.G.); chang.ye@stu.xjtu.edu.cn (Y.C.); 2 Department of Cardiac Ultrasound, the First Hospital of China Medical University, Shenyang 110001, China; junyang63@sina.com (J.Y.); rainbow3180@163.com (L.Z.); litan77cmuren@sina.com (T.L.)

**Keywords:** prevalence, risk factor, heart failure, preserved ejection fraction

## Abstract

*Background:* Heart failure with preserved ejection fraction (HFpEF) has attracted increasing attention worldwide. We aimed to estimate the prevalence of HFpEF and analyze its correlates in a sample of residents of northeast China; *Methods*: A population-based study of 2230 participants ≥35 years old was conducted in rural areas of Liaoning Province from January 2012 through August 2013. Information about lifestyle and other potential risk factors was obtained. HFpEF was diagnosed according to the recommendations of European Society of Cardiology; *Results*: The overall prevalence of HFpEF was 3.5% (1.8% in men and 4.9% in women). The prevalence of HFpEF increased with age in both genders and was greater in women than in men for every age group. Multivariable logistic regression analysis found that female gender (OR, 3.575; 95% CI, 1.761–7.256), hypertension (OR, 3.711; 95% CI, 2.064–6.674), and history of heart disease (2.086; 95% CI, 1.243–3.498) were associated factors for prevalent HFpEF; *Conclusions*: In a general population from rural northeast China, we found that female gender, hypertension, and history of heart disease were risk factors for prevalent HFpEF.

## 1. Introduction 

In the industrialized world, heart failure (HF) has been an important health concern and economic burden with an increasing prevalence and incidence [1,2]. According to the different pathophysiological mechanisms, heart failure is classified into two subtypes: heart failure with reduced ejection fraction (HFREF) and heart failure with preserved ejection fraction (HFpEF) [3]. Compared to patients with HFREF, patients with HFpEF have an increased morbidity and a similar or slightly lower mortality [4,5,6], but therapies that are beneficial for HFREF have failed to improve the outcomes of HFpEF [5,7]. Therefore, to better explore treatment options for HFpEF, it is essential to acquire more epidemiological data about HFpEF, such as its prevalence and related risk factors. Recent studies found that the prevalence of HFpEF varies greatly—from 13% to 74%—due to differences in diagnostic criteria and study population [8,9,10]. In general, the prevalence of HFpEF has increased yearly, and comprises 50% of all cases of heart failure [8]. One study of consecutive patients found that prevalence of HFpEF as the discharge diagnosis increased each year from 1987 to 2001, and the average prevalence increased from 38% to 47% to 54% in the three consecutive five-year periods included in the study [11]. The high prevalence and poor prognosis of HFpEF has attracted increasing attention. Recently, studies have identified several risk factors for HFpEF, such as age, hypertension, and the presence of ischemic heart disease [12,13,14]. Of note, most studies of the prevalence of HFpEF were conducted in selected populations, such as the elderly, inpatients or outpatients [11,15,16,17]; relatively few studies were conducted in a general population. Furthermore, most studies were conducted in the USA and Europe; hence, limited data is generalizable to Chinese populations, particularly rural residents of China. Therefore, epidemiological investigations in these areas are urgently needed.

This population-based epidemiological study aimed to estimate the prevalence and identify correlates of HFpEF in rural northeast China.

## 2. Methods

### 2.1. Study Population

From January 2012 through August 2013, a representative sample of residents of rural areas in Liaoning province aged ≥35 years was selected with multistage, stratified, random cluster sampling. In the first stage, one county (Liaoyang County) was randomly selected from rural areas of Liaoning Province. In the second stage, one town was randomly selected from Liaoyang County. In the third stage, eight rural villages from the selected town were randomly selected. Participants with pregnancy, malignant tumor, or a mental disorder were excluded from the present study. All eligible permanent residents aged ≥35 years from these villages were invited to participate in the study. In this study population, one out of three participants were randomly selected for echocardiography using a computer-generated random number table. The study was approved by the Ethics Committee of China Medical University (Shenyang, China, ethical approved project identification code: 2011-2-2). Written consent was obtained from all participants or authorized agents for those who were illiterate. 

### 2.2. Data Collection and Definitions

Information on covariates, such as age, gender, and lifestyle, were collected by cardiologists and trained nurses during a single clinic visit using a standard questionnaire by face-to-face interview. 

Family income was classified as ≤5000, 5000–20,000 or >20,000 CNY/year. Participants were asked whether they currently smoked or drank. Physical activity included occupational and leisure-time physical activity. A detailed description of the methods has been reported elsewhere [18]. Occupational and leisure-time physical activity were merged and regrouped into three categories: (1) low, participants who reported infrequent levels of both occupational and leisure-time physical activity; (2) moderate, participants who reported moderate or high levels of either occupational or leisure-time physical activity; and (3) high, participants who reported a moderate or high level of both occupational and leisure-time physical activity. Self-reported or family-reported histories of stroke or heart disease (including coronary heart disease, myocardial infarction, arrhythmia, and heart failure) were obtained from the questionnaire, and all participants who reported an incident of stroke or heart disease were asked for their permission to review their medical records.

Blood pressure was measured three times at 2-min intervals after at least 5 min rest using a standardized automatic electronic sphygmomanometer (HEM-907; Omron, Tokyo, Japan) according to American Heart Association protocol. The participants were asked to avoid caffeinated beverages and exercise for at least 30 min before the measurement. The mean of three results was calculated and used in all analyses. Heart rate was also measured with the electronic sphygmomanometer.

Weight and height were measured to the nearest 0.1 kg and 0.1 cm, respectively, with the participants wearing lightweight clothing and no shoes. Waist circumference (WC) was measured using a non-elastic tape (to the nearest 0.1 cm) at the level of the umbilicus at the end of normal expiration. Body mass index (BMI) was calculated as weight in kilograms divided by the square of the height in meters. 

Fasting blood samples were collected from all participants in the morning after at least 12 h of fasting. Blood samples, obtained from an antecubital vein, were collected into Vacutainer tubes containing ethylene diamine tetraacetic acid (EDTA). Fasting plasma glucose (FPG), total cholesterol (TC), low-density lipoprotein cholesterol (LDL-C), high-density lipoprotein cholesterol (HDL-C), triglyceride (TG), and other routine blood biochemical parameters were assayed enzymatically using an autoanalyzer. All laboratory equipment was calibrated, and blinded duplicate samples were used. 

According to the JNC-7 report [19], hypertension was defined as systolic blood pressure (SBP) ≥ 140 mm Hg and/or diastolic blood pressure (DBP) ≥ 90 mm Hg and/or use of antihypertensive medications. BMI values were categorized into three groups: normal (BMI < 25 kg/m^2^), overweight (25 ≤ BMI < 30 kg/m^2^), and obese (BMI ≥ 30 kg/m^2^), according to the World Health Organization (WHO) criteria [20]. Abdominal obesity was defined as WC ≥ 88 cm for females and WC ≥ 102 cm for males [21]. Dyslipidemia was defined according to the National Cholesterol Education Program-Third Adult Treatment Panel (ATP III) criteria [22]. High TC was defined as TC ≥ 6.21 mmol/L (240 mg/dL). Low HDL-C was defined as HDL-C < 1.03 mmol/L (40 mg/dL). High LDL-C was defined as LDL-C ≥ 4.16 mmol/L (160 mg/dL). High TG was defined as ≥2.26 mmol/L (200 mg/dL). Dyslipidemia was defined as having at least one of the following: high TC, high LDL-C, low HDL-C or high TG. Diabetes mellitus was diagnosed according to the WHO criteria [23]: FPG ≥ 7 mmol/L (126 mg/dL) and/or receiving treatment for diabetes. Glomerular filtration rate (GFR) was estimated using the Chronic Kidney Disease Epidemiology Collaboration (CKD-EPI) equation [24]. Reduced GFR was defined as an estimated GFR (eGFR) < 60 mL/min/1.73 m^2^. Anemia was defined as serum hemoglobin levels <13.0 g/dL (<130 g/L) for men and <12.0 g/dL (<120 g/L) for women, in accordance with WHO criteria [25]. Hyperuricemia was defined as uric acid levels >422 μmol/L for men and >363 μmol/L for women [26].

### 2.3. Echocardiography Measurements 

The echocardiograms were obtained using a commercially available Doppler echocardiograph (Vivid, GE Healthcare, Fairfield, CT, USA), with a 3.0-MHz transducer using M-mode, 2-dimensional, spectral Doppler and color Doppler transthoracic echocardiography with participants in the supine position. Three physicians specialized in echocardiography read and analyzed the echocardiograms. If any question or uncertainty arose, the other two specialists were consulted. The parasternal acoustic window was used to record two-dimensional and M-mode images of the left ventricular (LV) internal diameter, wall thickness, aortic root, and left atrium. The apical acoustic window was used to record four- and five-chamber images. Left ventricular mass (LVM) was calculated according to Devereux et al. [27]. LVM was divided by body surface area (BSA) to calculate the left ventricular mass index (LVMI). LV volumes were obtained from the apical four-chamber view, and the LV ejection fraction (LVEF) was calculated using the modified Simpson’s rule method [28]. LV end-diastolic volume was indexed for BSA. Doppler echocardiographic recordings were performed with the sample volume at the tips of the mitral valve leaflets in the apical four-chamber view. Peak early diastolic filling wave (E) velocity, peak atrial diastolic filling wave (A) velocity, and deceleration time (DT) were measured. Peak early (E’) and late (A’) diastolic mitral annular velocities were measured by pulsed wave tissue Doppler imaging (TDI) of the lateral wall in the apical four-chamber view.

**Figure 1:**
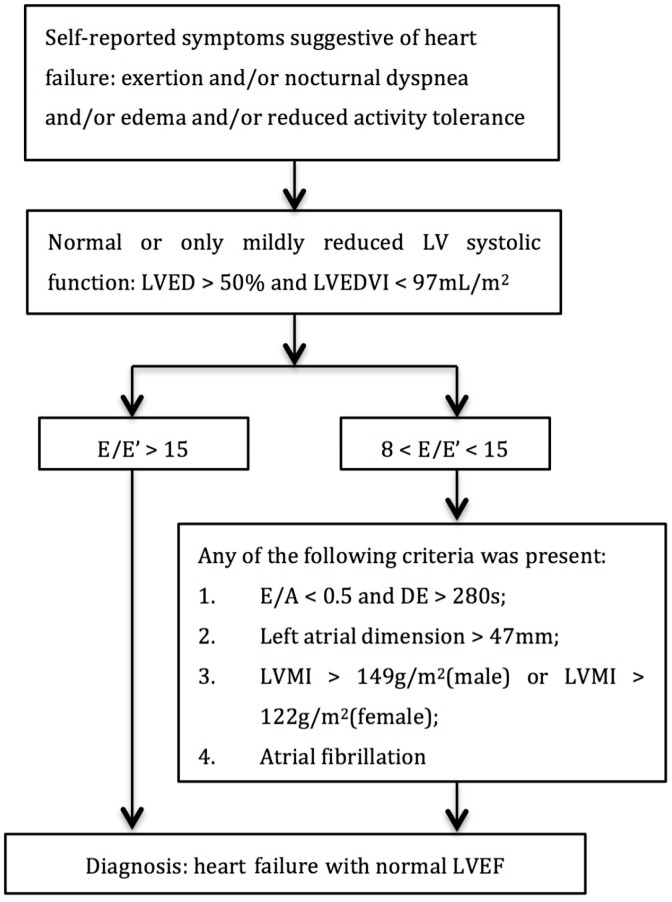
Diagnostic flowchart of heart failure with preserved ejection fraction (HFpEF). A: atrial diastolic filling wave velocity; E: early diastolic filling wave velocity; E’: early diastolic mitral annular velocity; LV: left ventricle; DE: E wave deceleration time; LVED: left ventricular end diastolic diameter; LVEDVI: left ventricular enddiastolic volume index; LVEF: left ventricular ejection fraction; LVMI: left ventricular mass index.

HFpEF was diagnosed according to the recommendation of the European Society of Cardiology (ESC) [29] with minor modifications. Participants with self-reported symptoms suggestive of heart failure (including exertional or nocturnal dyspnea, ankle swelling, and fatigue), normal, or only mildly reduced LV systolic function and reduced LV diastolic function were defined as HFpEF (Figure 1). As suggested by the consensus statement on the diagnosis of HFpEF in China [30] left atrial dimension was taken as one of the diagnostic criteria instead of left atrial volume index (LAVI). 

### 2.4. Statistical Analysis

Descriptive statistics were calculated for all variables; continuous variables were reported as mean values and standard deviations, and categorical variables as counts and percentages. Differences among categories were evaluated using non-parametric tests or the *χ*^2^-test, as appropriate. The Spearman rank correlation was employed for comparative analysis of the prevalence of HFpEF in different age groups. Multivariable logistic regression analyses were used to identify correlates of HFpEF; the strengths of associations were expressed as odds ratios (ORs) and corresponding 95% confidence intervals (CIs). All statistical analyses were performed using SPSS version 17.0 (SPSS Inc., Chicago, IL, USA), and *p* values < 0.05 were considered statistically significant. 

## 3. Results

### 3.1. Basic Characteristics of the Study Population

This study included 2230 participants (1055 males and 1175 females) aged ≥35 years. Table 1 shows the clinical and demographic characteristics of the study population according to sex. The mean age of the men and the women was 56 ± 11 and 55 ± 10 years, respectively. Although the mean BMI did not differ by gender, the mean waist circumference (WC) of men was larger than that of women (84.1 ± 10.0 cm and 82.1 ± 9.4 cm, respectively, *p* < 0.001). The physical activity of men was higher than that in women (*p* = 0.001). In addition, current smokers or drinkers were more common in men than in women (*p* < 0.001). Furthermore, the metabolism indicators in men tended to be more unfavorable (e.g., SBP, DBP, FPG, TC, LDL-C, and uric acid, all *p* < 0.01). The prevalence of two co-morbidities—abdominal obesity and history of heart disease—was considerably higher in women than that in men. 

### 3.2. Prevalence of HFpEF by Age and Sex

**Figure 2:**
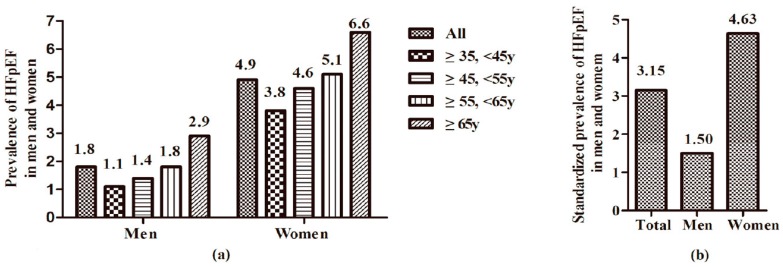
The (**a**) prevalence and (**b**) standardized prevalence of HFpEF in men and women.

HFpEF was diagnosed in 77 participants (19 men and 58 women), and the prevalence was 3.5% in the total study population. Prevalence of HEpEF is much higher in women than that in men (4.9% vs. 1.8%, *p* < 0.01). As shown in Figure 2a, in both sexes, the prevalence of HFpEF has a tendency to increase with age. The prevalence of HFpEF in men increased from 1.1% at 35–45 years to 2.9% at ≥65 years; for women, the prevalence increased from 3.8% in the youngest age group to 6.6% in the oldest age group. However, the Spearman rank correlation detected no significance for the correlation of the prevalence of HFpEF in different age groups in both sexes (*p* = 0.27 in men and *p* = 0.15 in women). Furthermore, in every age group, the prevalence of HFpEF was greater in women and was higher than that in men (*p* < 0.05). The standardized prevalence of HFpEF was 3.15% (1.50% in men and 4.63% in women), after being standardized by age according to the results of the 2010 Population Census of China [31] , as shown in Figure 2b. 

### 3.3. Prevalence of Clinical Co-Morbidities by Presence of HFpEF 

**Figure 3:**
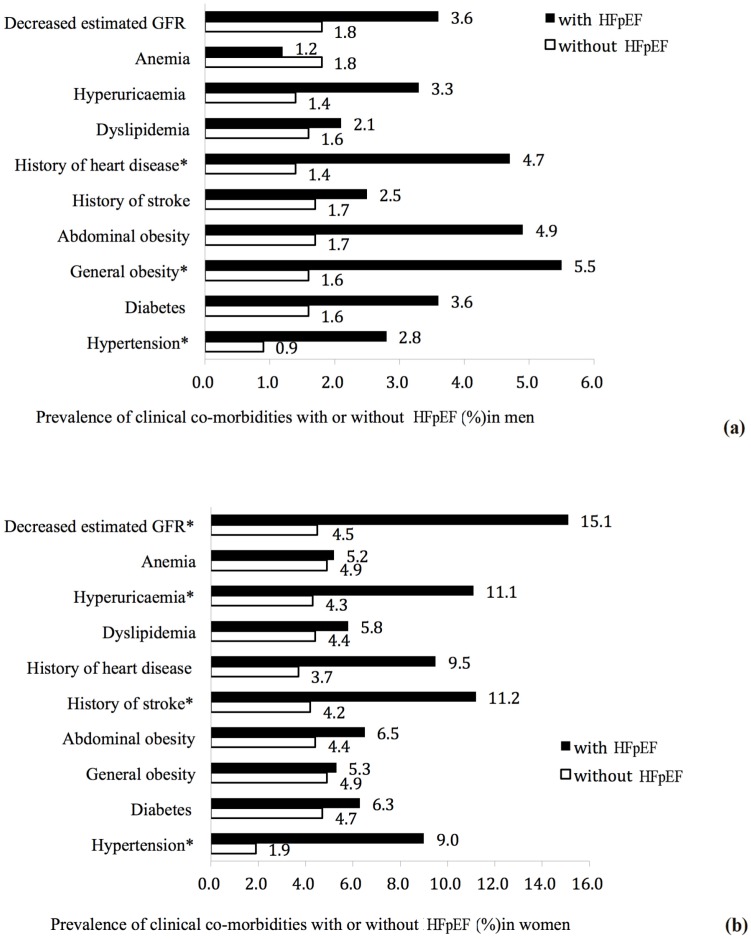
Prevalence of clinical co-morbidities with or without HFpEF (%) in men (**a**) and women (**b**). * *p* < 0.05, subjects with HFpEF vs. subjects without HFpEF.

Some clinical co-morbidities, including hypertension, diabetes, general obesity, abdominal obesity, history of stroke, history of heart disease, dyslipidemia, hyperuricemia, anemia, and decreased estimated GFR, were analyzed for a correlation with HFpEF. As shown in Figure 3a,b, men with HFpEF tended to have a higher prevalence of hypertension (2.8% vs. 0.9%, *p* = 0.02), general obesity (5.5% vs. 1.6%, *p* = 0.036), history of heart disease (4.7% vs. 1.4, *p* = 0.009), and women with HFpEF tended to have a higher prevalence of hypertension (9.0% vs. 1.9%, *p* < 0.001), history of stroke (11.2% vs. 4.2%, *p* = 0.001), hyperuricemia (11.1% vs. 4.3%, *p* = 0.001), and decreased estimated GFR (15.1% vs. 4.5%, *p* < 0.001).

### 3.4. Factors Associated with HFpEF

Results of multiple logistic regression analysis showed that hypertension and history of heart disease were associated factors for the prevalence of HFpEF in women (Table 2). In women, those with a history of hypertension and heart disease were more likely to have HFpEF, compared with those with normal blood pressure and those without heart disease (hypertension: OR, 4.462; 95% CI, 2.222–8.960, *p* < 0.001; history of heart disease: OR, 1.872; 95% CI, 1.034–3.390, *p* = 0.039). 

## 4. Discussion

This study revealed for the first time that the prevalence of HFpEF in the rural population of northeast China was 3.5%. We observed a higher prevalence of HFpEF in women than that in men (4.9% vs. 1.8%, respectively). The participants with female gender, hypertension, or history of heart disease tended to have a higher prevalence of HFpEF.

With the variation of the definition and diagnostic criteria of heart failure, the prevalence of HFpEF varied greatly in different studies [8,10,32]. The differences of study population, geographic area, and the year of data collection may also contribute to the prevalence of HFpEF [15]. A population-based cohort study found that the patients with heart failure comprised approximately 5% of the total population [33], and about half of the patients with heart failure had preserved normal left ventricular ejection fraction [34,35]. Our results were in accordance with these findings. However, because the prevalence of HFpEF in general populations in China are not reported, we could not determine whether our results agreed with the prevalence of HFpEF in other regions of China. 

In general, most studies have concluded that age is an important risk factor for HFpEF [3]. We found that the prevalence of HFpEF was 4.8% in participants over 65 years old, which was consistent with the result of a study conducted in central Italy that found a 4.9% HFpEF prevalence in 65–84 year-old individuals [15]. Hedberg et al. [36] reported that the prevalence of HFpEF in a population-based sample of 75-year-old participants was 6.8%. The Olmsted County study [37] found that the prevalence of HFpEF was 13% in a population aged over 75 years, and the UK ECHOES study [38] found that it was 17% in a population aged over 85 years. In our study, we could still see a tendency that the prevalence of HFpEF increased with age; however, Spearman rank correlation detected no significance between increasing age and the prevalence of HFpEF. The low prevalence of HFpEF and the small sample size of the study may be the reason. However, the high proportion of hypertension and other cardiovascular risk factors in the elderly population [39] may influence the correlation results. On one hand, the prevalence of the cardiovascular risk factors increases with age; on the other hand, the prevalence of cardiovascular risk factors is associated with HFpEF. Therefore, although there was a noticeable increase of HFpEF with age, Binary Logistic regression did not reveal such an association. 

Regarding gender, in the present study, we found that women had a higher prevalence of HFpEF, which was in accordance with another study that found the age-standardized prevalence of HFpEF for women and men was 5.1% and 3.0%, respectively [39]. The Rotterdam Study reported that the overall prevalence of heart failure was 3.9% and did not differ between men and women, but the prevalence of left ventricular systolic dysfunction (HFREF) was higher in men than in women [40]. Frank P. Brouwers et al. [3] found that female gender, atrial fibrillation, higher cystatin C, and urinary albumin excretion were particularly strong predictors for HFpEF. This is possibly because women are more likely to suffer from metabolic syndrome, which is characterized by hyperlipidemia, hypertension, diabetes mellitus, abdominal obesity [41], and stroke [42]. These diseases could increase the risk of HFpEF in women, as previously mentioned.

Our study found that participants with clinical comorbidities such as history of heart disease and hypertension had a higher prevalence of HFpEF, consistent with the results from other studies [3,11,43,44,45]. As demonstrated by a previous study [3], people with a previous myocardial infarction have an increased risk specifically for new-onset HFpEF. Of note, renal dysfunction and cardiorenal syndrome were common in patients with HFpEF, which might be related to protracted fluid retention and refractory hypertension [41]. Unlike the unalterable factors, such as age and gender, an appropriate treatment of the comorbidity could be crucial for the prevention of HFpEF. For example, hypertension is generally considered to lead to the development of HFpEF [45], which is consistent with our results. Hence, early diagnosis and treatment of hypertension was proven to be effective for the prevention of HFpEF. The above strategy may also apply to other comorbidities, such as decreased eGFR, anemia, hyperuricemia, dyslipidemia, obesity, and diabetes mellitus.

In our study, some limitations should be mentioned. Brain natriuretic peptide (BNP) or N-terminal Pro-brain Natriuretic Peptide (NTproBNP) was not checked because of a limited budget in our study. Participants with pregnancy, malignant tumor, and mental disorder were excluded from the present study, which influenced participation in our survey and the prevalence of HFpEF. Furthermore, the participants in our study were limited to the rural areas of Liaoning Province in northeast China, so our findings cannot be generalized to other regions of China. Other investigations of the general population of urban residents and southern rural areas are needed to validate our findings and to estimate the prevalence of HFpEF throughout China. 

## 5. Conclusions

In a general population-based study in rural northeast China, we found that the overall prevalence of HEPEF was 3.5% (4.9% in women and 1.8% in men). Participants with hypertension and history of heart disease tended to have a higher risk for HFpEF. The prevalence of HEPEF increased with age in both sexes, and females were more likely to have HEPEF in every age group. 

## Figures and Tables

**Table 1:** Baseline characteristics of the study population.

Variables	Total (*n* = 2230)	Male (*n* = 1055)	Female (*n* = 1175)	*p*-Value
**Demographics**				
Age (years)	56 ± 11	56 ± 11	55 ± 10	0.025
Current smoking status	818 (36.7)	597 (56.6)	221 (18.8)	<0.001
Current drinking status	440 (19.7)	421 (39.9)	19 (1.6)	<0.001
Physical activity				0.001
Low	509 (22.8)	205 (19.4)	304 (25.9)	
Moderate	1540 (69.1)	762 (72.2)	778 (66.2)	
High	181 (8.1)	88 (8.3)	93 (7.9)	
Family income (CNY/year)				0.345
≤5000	427 (19.1)	213 (20.2)	214 (18.2)	
5000–20,000	1196 (53.6)	550 (52.1)	646 (55.0)	
>20,000	607 (27.2)	292 (27.7)	315 (26.8)	
BMI (kg/m^2^)	24.2 ± 3.6	24.1 ± 3.6	24.3 ± 3.7	0.207
WC (cm)	83.1 ± 9.7	84.1 ± 10.0	82.1 ± 9.4	<0.001
SBP (mm Hg)	137.9 ± 23.0	139.7 ± 22.1	136.3 ± 23.7	<0.001
DBP (mm Hg)	81.1 ± 11.6	82.6 ± 11.2	79.7 ± 11.8	<0.001
**Laboratory variables**				
TC (mmol/L)	5.13 ± 1.06	5.05 ± 1.06	5.20 ± 1.05	0.001
TG (mmol/L)	1.78 ± 1.73	1.84 ± 2.00	1.73 ± 1.44	0.143
LDL-C (mmol/L)	2.67 ± 0.67	2.63 ± 0.64	2.71 ± 0.69	0.005
HDL-C (mmol/L)	1.32 ± 0.30	1.30 ± 0.33	1.33 ± 0.27	0.034
FPG (mmol/L)	5.95 ± 1.63	6.00 ± 1.64	5.91 ± 1.63	0.220
Uric acid (μmol/L)	315.4 ± 87.8	356.7 ± 88.0	278.3 ± 69.2	<0.001
Hemoglobin (g/L)	136.5 ± 14.8	146.3 ± 12.7	127.8 ± 10.5	<0.001
Estimated GFR (mL/min/1.73 m^2^)	87.5 ± 14.6	90.2 ± 13.8	85.1 ± 14.8	<0.001
**Echocardiographic parameters**				
LAD, cm	3.4 ± 0.4	3.5 ± 0.4	3.3 ± 0.4	<0.001
LVEDV, mL	104.3 ± 23.9	112.7 ± 22.4	96.7 ± 22.5	<0.001
LVESV, mL	37.2 ± 10.2	39.6 ± 11.4	35.0 ± 8.6	<0.001
LVEF, %	64.1 ± 6.8	64.9 ± 5.9	63.4 ± 7.4	<0.001
E/E′	9.1 ± 3.9	8.6 ± 3.7	9.5 ± 4.1	<0.001
E/A	1.0 ± 0.6	1.0 ± 0.7	1.0 ± 0.4	0.252
EDT, ms	191.8 ± 37.7	191.1 ± 35.7	192.4 ± 39.4	0.337
LVMI, g/m^2^	90.3 ± 57.5	95.3 ± 67.4	85.7 ± 46.5	<0.001
**Co-morbidities**				
Hypertension	1001 (44.9)	499 (47.3)	502 (42.7)	0.03
Diabetes	253 (11.3)	110 (10.4)	143 (12.2)	0.195
General obesity	131 (5.9)	55 (5.2)	76 (6.5)	0.208
Abdominal obesity	351 (15.7)	41 (3.9)	310 (26.4)	<0.001
History of stroke	244 (10.9)	119 (11.3)	125 (10.6)	0.628
History of heart disease	370 (16.6)	129 (12.2)	241 (20.5)	<0.001
Dyslipidemia	856 (38.4)	424 (40.2)	432 (36.8)	0.097

Data are expressed as the mean ± SD or as *n* (%). Abbreviations: CNY, China Yuan (1 CNY = 0.161 USD); BMI, body mass index; WC, waist circumference; SBP, systolic blood pressure; DBP, diastolic blood pressure; TC, total cholesterol; TG, triglyceride; LDL-C, low-density lipoprotein cholesterol; HDL-C, high-density lipoprotein cholesterol; FPG, fasting plasma glucose; GFR, glomerular filtration rate. LAD, left atrial diameter; LVESV, left ventricular endsystolic volume; EDT, E wave deceleration time; Dyslipidemia was defined as having at least one of the following: high TC, high LDL-C, low HDL-C or high TG.

**Table 2:** Results of multivariable regression analyses of potential risk factors for heart failure with preserved ejection fraction.

Variable	Men			Women		
	OR	95% CI	*p*-Value	OR	95% CI	*p*-Value
Age (year)	1.016	0.966–1.070	0.536	0.973	0.939–1.008	0.128
Current smoking status	0.671	0.254–1.775	0.422	1.713	0.902–3.253	0.1
Current drinking status	1.455	0.549–3.854	0.45	2.007	0.420–9.592	0.383
Physical activity	1.156	0.492–2.716	0.74	0.916	0.562–1.493	0.725
Dyslipidemia	0.863	0.307–2.423	0.78	0.902	0.498–1.637	0.735
Hyperuricaemia	1.99	0.697–5.684	0.199	1.755	0.815–3.777	0.15
Anemia	0.673	0.080–5.694	0.716	1.234	0.583–2.613	0.583
Decreased estimated GFR	0.908	0.089–9.245	0.935	1.983	0.723–5.435	0.183
Hypertension	2.346	0.784–7.018	0.127	4.462	2.222–8.960	<0.001 *
Diabetes	1.799	0.543–5.958	0.337	0.759	0.340–1.695	0.501
General obesity	1.911	0.284–12.867	0.505	0.75	0.234–2.403	0.628
Abdominal obesity	1.076	0.119–9.719	0.948	1.152	0.611–2.170	0.662
History of stroke	1.12	0.297–4.220	0.868	1.913	0.951–3.845	0.069
History of heart disease	2.602	0.917–7.388	0.072	1.872	1.034–3.390	0.039 *
Income	1.031	0.502–2.120	0.934	1.048	0.667–1.647	0.839

* *p* < 0.05 Factor is independently associated with heart failure with preserved LVEF while adjusting for the remaining factors. Abbreviations: LVEF, left ventricular ejection fraction; OR, odds ratio; 95% CI, 95% confidence interval; CNY, China Yuan (1 CNY = 0.161 USD); TC, total cholesterol; TG, triglyceride; LDL-C, low-density lipoprotein cholesterol; HDL-C, high-density lipoprotein cholesterol; GFR, glomerular filtration rate.
